# Surgery for acute type A aortic dissection

**DOI:** 10.1093/icvts/ivaf028

**Published:** 2025-02-13

**Authors:** Tirone E David

**Affiliations:** Division of Cardiovascular Surgery of the Peter Munk Cardiac Centre, Toronto General Hospital and University of Toronto, Toronto, ON, Canada

**Keywords:** aortic aneurysm, aortic dissection, aortic surgery

In this issue of this Journal, Timmermans *et al*. [1] from the Netherlands reported on the outcomes of surgery for type A aortic dissection in 1357 patients operated on from 2018 through 2021. The authors were able to determine post-discharge survival and cause of death in 1317 patients by linking this cohort with the country’s vital statistics, an administrative database. The operative mortality fell from 20.4% in 2018 to 13.9% in 2022 and the number of patients who had surgery increased during that period of observation. The authors did not provide the number of patients with type A aortic dissection who were not operated on, the timing of surgery with the start of the dissection or an analysis of risk factors for operative mortality. Regardless, an overall operative mortality of 13.9% for all patients is commendable.

Why can’t we reduce the operative mortality for acute type A aortic dissection (ATAAD)? The International Registry of Acute Aortic Dissection (IRAD) was the first multicentre database created, and it has provided valuable information on the management of this disorder and the risk factors associated with surgical treatment [2, 3]. Older age, previous aortic valve replacement, cardiogenic shock, any pulse deficit and myocardial infarction are associated with higher operative mortality [3]. Age alone should not be a contraindication for surgery [4]. In the IRAD database, the proportion of octogenarians who had surgery was lower than in septuagenarians (68.1% vs 85.9%, *P* < 0.001), and with higher mortality (32.0% vs 25.6%, *P* = 0.008) [4]. It is not surprising that surgery for ATAAD in patients who had previous aortic valve replacement is associated with higher risk [5]. Cardiogenic shock increased operative mortality when compared with haemodynamically stable patients (30.2% vs 23.9%, *P* = 0.007) in the IRAD database [6]. ATAAD may be misdiagnosed in patients presenting with acute myocardial infarction and may delay surgical treatment, increasing the operative risk. The electrocardiogram showed ischaemic changes in 15% of patients, and evidence of acute myocardial infarction in 5% in the IRAD database [2]. This preoperative finding was associated with higher mortality [3]. Organ malperfusion is probably the most serious complication of this disorder and depending on which organs are involved, the operative mortality increases dramatically as well as late mortality [7, 8]. There is general agreement that patients with ATAAD should be operated on as an emergency and as soon as the diagnosis is made, especially if there is evidence of malperfusion [7, 8]. The re-establishment of blood flow into the true lumen as soon as possible reduces operative mortality, largely because of reduced postoperative complications related to malperfusion [7, 8]. If malperfusion persists after replacement of the proximal aorta, further intervention may be necessary [7]. Patients with advanced mesenteric ischaemia may be best treated with fenestration and stenting before proximal aortic repair, but mortality in this sub-group is very high [9]. There is no uniform consensus on what approach is best to treat patients with preoperative evidence of mesenteric ischaemia and the treatment should be individualized [7, 9]. In the German Registry for Acute Aortic Dissection, the overall 30-day mortality in 2137 consecutive patients was 16.9%, and one-third of them had preoperative evidence of malperfusion [10]. If there was no malperfusion, the mortality was 12.6%, whereas if one system was malperfused was 21.3%, 2 systems 30.9% and 3 systems 43.4% (*P* < 0.001) [10].

An important variable that has not been analysed is the influence of the surgeon on the outcome of surgery for ATAAD. Surgery performed by an experienced aortic surgeon on a haemodynamically stable patient without malperfusion should have operative mortality in low single digits. A recent report on surgery for ATAAD in 467 patients from the University of Pittsburgh indicated an operative mortality of 5.7% in patients without malperfusion and 21.5% in patients with malperfusion [7]. Unfortunately, in a disease that carries hourly mortality of ∼2%, referral to an aortic centre may reduce operative mortality but increase overall mortality of ATAAD by delaying surgery [8].
